# Supplementary material for: Nutrient-Level Evaluation of Meals Provided on the Government-Funded School Lunch Program in New Zealand
Source: Nutrients. 2022 Nov 30;14(23):5087. doi: 10.3390/nu14235087 (PMC9741039; doi:10.3390/nu14235087)
Supplement: Supplementary file 1 [file nutrients-14-05087-s001.zip › nutrients-2039245-supplementary.pdf]

Supplementary Material

Supplementary Table S1. Percentage of meals above a third of daily requirements

	Years 0-3 meals				Years 4-8 meals				Years 9+ meals			
**Nutrients (% RDI)**	**5-year-olds**		**8-year-olds**		**9-year-olds**		**12-year-olds**		**13-year-olds**		**18-year-olds**	
	female	male	female	male	female	male	female	male	female	male	female	male
Energy (kj)	11.0	11.0	8.0	4.0	9.3	3.1	1.0	0.0	5.7	3.8	3.8	0.0
Protein	100.0	100.0	100.0	100.0	97.9	97.9	97.9	97.9	99.1	99.1	98.1	85.7
Thiamine	57.0	57.0	57.0	57.0	67.0	40.2	67.0	40.2	84.8	72.4	84.8	47.6
Fiber[1]	79.0	79.0	79.0	79.0	63.9	63.9	63.9	63.9	79.1	79.1	61.9	50.5
Riboflavin	64.0	64.0	64.0	64.0	47.4	47.4	47.4	47.4	67.6	67.6	43.8	28.6
Niacin equivalents	99.0	99.0	99.0	99.0	95.9	95.9	95.9	95.9	100.0	100.0	100.0	100.0
Vitamin C	65.0	65.0	65.0	65.0	58.8	58.8	58.8	58.8	75.2	75.2	75.2	75.2
Vitamin B6	80.0	80.0	80.0	80.0	46.4	46.4	46.4	46.4	68.6	68.6	51.4	46.7
Vitamin B12	66.0	66.0	66.0	66.0	59.8	59.8	59.8	59.8	64.8	64.8	55.2	55.2
Folate -dietary folate equivalents	78.0	78.0	78.0	78.0	67.0	67.0	67.0	67.0	74.3	74.3	65.7	65.7

Vitamin A equivalents	87.0	87.0	87.0	87.0	73.2	73.2	73.2	73.2	79.1	79.1	74.3	61.9
Magnesium	88.0	88.0	88.0	88.0	46.4	46.4	46.4	46.4	79.1	79.1	32.4	20.0
Calcium	33.0	33.0	33.0	33.0	3.1	3.1	3.1	3.1	12.4	12.4	12.4	12.4
Iron	19.0	19.0	19.0	19.0	55.7	55.7	55.7	55.7	76.2	76.2	8.6	37.1
Zinc	91.0	91.0	91.0	91.0	70.1	70.1	70.1	70.1	87.6	87.6	77.1	24.8
Selenium	72.0	72.0	72.0	72.0	45.4	45.4	45.4	45.4	71.4	71.4	58.1	45.7
Iodine	31.0	31.0	31.0	31.0	32.0	32.0	32.0	32.0	35.2	35.2	28.6	28.6

Notes: [1]Adequate intake

Supplementary Table S2. Comparisons of nutrients in years 0-3 Ka Ora, Ka Ako meals to international nutrient-level standards

		Age (years)	Energy (kJ)	Protein g (% of energy)	Carbohydrate g (% of energy)	Fat g (% of energy)	Saturated Fat g (% of energy)	Fibre g	Sodium mg
**Ka Ora, Ka Ako**		**5-8**	**1522**	**20.4 (22.4)**	**39.6 (43.5)**	**12.6 (31.2)**	**4.3 (10.5)**	**6.4**	**502**
England[1]		5-11	2215 ± 5%	≥7.5	≥70.6	≤20.6	≤6.5	≥4.2	≤499
Wales[2]		5-11	2215 ± 5 %	≥7.5	≥70.6	≤20.6	≤6.5	≥4.2	≤499
Finland[3]		6-9	2299	(13-17)	(45-50)	(30-40)	(≤10)		
Sweden[4]		6-9	2090	12-24 (10-20)	55-73 (45-60)	14-22 (25-40)	≤6 (≤10)	≥6	≤480
China[5]		6-11	2596	≥17.4	(50-65)	(20-30)			
USA (CNR meal guidelines)[6]			2780	≥10		(≤30)	(≤10)		
USA (HHFKA meal guidelines)[7]		5-11	2299-2717	≥15.2		(≤30)	(≤10)	≥8.5	≤640
Japan[8]		6-7	2215	16-26		(25-30)		≥4	≤800
		8-9	2675	18-32		(25-30)		≥5	≤1000
Korea[9]	Males	7-9	2232	≥8.4					
	Females	7-9	2090	≥8.4					
Taiwan[9]		7-9	2801	≥22		≤20			≤800

Supplementary Table S3. Comparisons of nutrients in years 0-3 Ka Ora, Ka Ako meals to international nutrient-level standards (continued)

		Age (years)	Vitamin A (µg)	Vitamin C (mg)	Thiamine (µg)	Riboflavin (µg)	Folate (µg)	Calcium (mg)	Iron (mg)	Zinc (mg)
**Ka Ora, Ka Ako**		**5-8**	**324**	**14.9**	**270**	**230**	**118**	**181**	**2.3**	**2.1**
England[1]		5-11	≥175	≥10.5			≥53	≥193	≥3	≥2.5
Wales[2]		5-11	≥175	≥10.5			≥53	≥193	≥3	≥2.5
Sweden[4]		6-9	≥120	≥12	≥270	≥330	≥39	≥210	≥2.7	≥2.1
China[5]		6-11	≥199	≥26	≥390	≥390		≥390	≥5.14	≥2.81
USA (CNR meal guidelines)[6]			≥224	≥15				≥286	≥3.5	
USA (HHFKA meal guidelines)[7]		5-11	≥192	≥24				≥332	≥3.4	
Japan[8]		6-7	≥150	≥20	≥300	≥400		≥300	≥2	≥2
		8-9	≥170	≥20	≥400	≥400		≥350	≥3	≥2
Korea[9]	Males	7-9	≥97	≥13.4	≥200	≥240		≥184	≥2.4	
	Females	7-9	≥90	≥13.4	≥170	≥200		≥184	≥2.4	
Taiwan[9]		7-9						≥270		

Supplementary Table S4. Comparisons of nutrients in years 4-8 Ka Ora, Ka Ako meals to international nutrient-level standards

	Age (years)	Energy (kJ)	Protein g (% of energy)	Carbohydrate g (% of energy)	Fat g (% of energy)	Saturated Fat g (% of energy)	Fibre g	Sodium mg
**Ka Ora, Ka Ako**	**9-12**	**1772**	**22.2 (20.9)**	**49.8 (47.0)**	**13.0 (27.6)**	**4.8 (10.2)**	**7.7**	**595**
England[1]	5-11	2215 ± 5%	≥7.5	≥70.6	≤20.6	≤6.5	≥4.2	≤499
Wales[2]	5-11	2215 ± 5%	≥7.5	≥70.6	≤20.6	≤6.5	≥4.2	≤499
Finland[3]	10-13	2926	(13-17)	(45-50)	(30-40)	(≤10)		
Sweden[4]	10-12	2696	16-32 (10-20)	71-95 (45-60)	18-29 (25-40)	≤7 (≤10)	≥8	≤720
China[5]	12-15	3273	≥23.4	(50-65)	(20-30)			
Slovenia[10]	10-13	2633-3072	15-28 (10-15)	≥78 (>50)	21-29 (30-35)	≤3 (≤10)	≥6	≤720
India[11]	6-14	1881	≥12					
USA (CNR meal guidelines)[6]		2780	≥10		(≤30)	(≤10)		
USA (HHFKA meal guidelines)[7]	11-14	2508-2926				(≤10)		≤710
Japan[8]	10-11	3135	22-38		(25-30)		≥6	≤1000

Supplementary Table S5. Comparisons of nutrients in years 4-8 Ka Ora, Ka Ako meals to international nutrient-level standards (continued)

	Age (years)	Vitamin A (µg)	Vitamin C (mg)	Thiamine (µg)	Riboflavin (µg)	Folate (µg)	Calcium (mg)	Iron (mg)	Zinc (mg)
**Ka Ora, Ka Ako**	**9-12**	**337**	**16.6**	**380**	**270**	**184**	**216**	**2.9**	**2.5**
England[1]	5-11	≥175	≥10.5			≥53	≥193	≥3	≥2.5
Wales[2]	5-11	≥175	≥10.5			≥53	≥193	≥3	≥2.5
Sweden[4]	10-12	≥180	≥15	≥330	≥390	≥60	≥270	≥3.3	≥3.3
China[5]	12-15	≥258	≥36	≥500	≥480		≥413	≥6.3	≥3.7
Japan[8]	9-11	≥200	≥25	≥500	≥500		≥400	≥4	≥3
USA (CNR meal guidelines)[6]		≥224	≥15				≥286	≥3.5	
USA (HHFKA meal guidelines)[7]	11-14	≥192	≥24				≥332	≥3.4	

Supplementary Table S6. Comparisons of nutrients in years 9+ Ka Ora, Ka Ako meals to international nutrient-level standards

	Age (years)	Energy (kJ)	Protein g (% of energy)	Carbohydrate g (% of energy)	Fat g (% of energy)	Saturated Fat g (% of energy)	Fibre g	Sodium mg
**Ka Ora, Ka Ako**	**13-18**	2332	31.9 (22.9)	58.3 (41.8)	17.3 (27.9)	6.3 (10.2)	9.0	825
England[1]	11-18	2700 ± 5%	≥13.3	≥86.1	≤25.1	≤7.9	≥5.2	≤714
Wales co-ed schools[2]	11-18	2700 ± 5%	≥13.3	≥86.1	≤25.1	≤7.9 (≤10)	≥5.2	≤714
Finland[3]	14-16	3553	(13-17)	(45-50)	(30-40)	(≤10)		
Sweden[4]	13-15	3072	18-36 (10-20)	81-108 (45-60)	21-33 (25-40)	≤8 (≤10)	≥9	≤720
	16-18	3302	19-39 (10-20)	87-116 (45-60)	22-36 (25-40)	≤9 (≤10)	≥10	≤720
China[5]	15-18	3528	≥25.3	(50-65)	(20-30)			
India[11]	14-18	2926	≥20					
USA (HHFKA meal guidelines)[7]	14-18	3135-3553				(≤10)		≤740
Japan[8]	12-14	3428	25-40		(25-30)			≤1200

Supplementary Table S7. Comparisons of nutrients in years 9+ Ka Ora, Ka Ako meals to international nutrient-level standards (continued)

	Age (years)	Vitamin A (µg)	Vitamin C (mg)	Thiamin (µg)	Riboflavin (µg)	Folate (µg)	Calcium (mg)	Iron (mg)	Zinc (mg)
**Ka Ora, Ka Ako**	**13-18**	**375**	**23.5**	**400**	**350**	**177**	**256**	**3.3**	**3.2**
England[1]	11-18	≥245	≥14			≥70	≥350	≥5.2	≥3.3
Sweden[4]	13-15		≥23			≥90		≥4.5	
	16-18		≥23			≥120		≥4.5	
Wales - co-ed schools[2]	11-18	≥245	≥14			≥70	≥300	≥4.4	≥2.8
China[5]	15-18	≥272	≥38	≥540	≥510		≥375	≥6.4	≥3.8
Japan[8]	12-14	≥300	≥35	≥500	≥600		≥450	≥4	≥3

Supplementary Table S8. Key sources of sodium in Ka Ora, Ka Ako meals

**Sodium**
Wholemeal bread products (>18.6%*)
Sauces (>17.6%*)
Cheese (14.1%)
Processed meat products (>13.6%*)

*Calculations include all products contributing ≥0.2%

References

1. United Kingdom Parliament. *The Education (Nutritional Standards and Requirements for School Food) (England) Regulations 2007*. (Queen's Printer of Acts of Parliament, 2007).

2. Department for Education and Skills. *Healthy eating in maintained schools: Statutory guidance for local authorities and governing bodies*. www.legislation.gov.uk/wsi/2013/1984/contents/made (2014).

3. National Nutrition Council. *Eating and Learning Together: recommendations for school meals*. http://urn.fi/urn:iSBn:978-952-302-844-9 (2017).

4. The National Food Agency Sweden. *Good school meals. Guidelines for primary schools, secondary schools and youth recreation centres*. http://docplayer.net/16531972-Good-school-meals-guidelines-for-primary-schools-secondary-schools-and-youth-recreation-centres.html (2013).

5. Huang, Z. *et al.* Food and Nutrients Intake in the School Lunch Program among School Children in Shanghai, China. *Nutr. 2017, Vol. 9, Page 582* **9**, 582 (2017).

6. Smith, S. L. & Cunningham-Sabo, L. Food choice, plate waste and nutrient intake of elementary- and middle-school students participating in the US National School Lunch Program. *Public Health Nutr.* **17**, 1255–1263 (2014).

7. United States of America Department of Agriculture. *Nutrition Standards in the National School Lunch and School Breakfast Programs; Final Rule. Federal Register* (2012).

8. Morimoto, K. & Miyahara, K. Nutritional Management Implemented at School Lunch Programs in Japan Based on the Changes in Criteria for Provision of School Lunches. *Japanese J. Nutr. Diet.* **76**, S23–S37 (2018).

9. Kim, M. *et al.* Comparison of the nutrient-based standards for school lunches among South Korea, Japan, and Taiwan. *Asia Pac. J. Clin. Nutr.* **26**, 160–168 (2017).

10. Poličnik, R. *et al.* Energy and Nutritional Composition of School Lunches in Slovenia: The Results of a Chemical Analysis in the Framework of the National School Meals Survey. *Nutr. 2021, Vol. 13, Page 4287* **13**, 4287 (2021).

11. El-Rifai, S. March/April 2020 Food Services Newsletter. *Framingham State University* https://campussuite-storage.s3.amazonaws.com/prod/11162/b2004386-1ca3-11e6-b537-22000bd8490f/2054981/32c82f80-571c-11ea-b524-12f4a7a23217/file/FSindiaedit.pdf (2020).
